# Spatiotemporal Modeling of Ozone Levels in Quebec (Canada): A Comparison of Kriging, Land-Use Regression (LUR), and Combined Bayesian Maximum Entropy–LUR Approaches

**DOI:** 10.1289/ehp.1306566

**Published:** 2014-05-30

**Authors:** Ariane Adam-Poupart, Allan Brand, Michel Fournier, Michael Jerrett, Audrey Smargiassi

**Affiliations:** 1 Department of Environmental and Occupational Health, Faculty of Public Health, Université de Montréal, Montréal, Québec, Canada; 2 Institut national de santé publique du Québec (INSPQ), Montréal, Québec, Canada; 3 Direction de santé publique de Montréal, Montréal, Québec, Canada; 4 Department of Environmental Health, University of California, Berkeley, Berkeley, California, USA; 5 Chaire sur la pollution de l’air, les changements climatiques et la santé, Department of Environmental and Occupational Health, Faculty of Public Health, Université de Montréal, Montréal, Québec, Canada; * These authors contributed equally to this work.

## Abstract

Background: Ambient air ozone (O_3_) is a pulmonary irritant that has been associated with respiratory health effects including increased lung inflammation and permeability, airway hyperreactivity, respiratory symptoms, and decreased lung function. Estimation of O_3_ exposure is a complex task because the pollutant exhibits complex spatiotemporal patterns. To refine the quality of exposure estimation, various spatiotemporal methods have been developed worldwide.

Objectives: We sought to compare the accuracy of three spatiotemporal models to predict summer ground-level O_3_ in Quebec, Canada.

Methods: We developed a land-use mixed-effects regression (LUR) model based on readily available data (air quality and meteorological monitoring data, road networks information, latitude), a Bayesian maximum entropy (BME) model incorporating both O_3_ monitoring station data and the land-use mixed model outputs (BME-LUR), and a kriging method model based only on available O_3_ monitoring station data (BME kriging). We performed leave-one-station-out cross-validation and visually assessed the predictive capability of each model by examining the mean temporal and spatial distributions of the average estimated errors.

Results: The BME-LUR was the best predictive model (*R*^2^ = 0.653) with the lowest root mean-square error (RMSE ;7.06 ppb), followed by the LUR model (*R*^2^ = 0.466, RMSE = 8.747) and the BME kriging model (*R*^2^ = 0.414, RMSE = 9.164).

Conclusions: Our findings suggest that errors of estimation in the interpolation of O_3_ concentrations with BME can be greatly reduced by incorporating outputs from a LUR model developed with readily available data.

Citation: Adam-Poupart A, Brand A, Fournier M, Jerrett M, Smargiassi A. 2014. Spatiotemporal modeling of ozone levels in Quebec (Canada): a comparison of kriging, land-use regression (LUR), and combined Bayesian maximum entropy–LUR approaches. Environ Health Perspect 122:970–976; http://dx.doi.org/10.1289/ehp.1306566

## Introduction

Tropospheric ozone (O_3_) is a photochemical pollutant that has increased globally in concentration since the 19th century (Bogaert et al. 2009). Short- and long-term exposure to ambient O_3_ has been associated with a variety of adverse health outcomes, including respiratory, cardiovascular, and neurological conditions and, possibly, increased mortality [Chen et al. 2007; Institut national de recherche et de sécurité pour la prévention des accidents du travail et des maladies professionnelles 1997; Jerrett et al. 2009; U.S. Environmental Protection Agency (EPA) 2006].

Large population studies designed to assess the health risks of O_3_ exposure require accurate exposure estimates. The assessment of the exposure of a population is a complex task because O_3_ exposure exhibits complex spatiotemporal patterns, which present considerable modeling challenges. Worldwide, modeling methods have been developed to improve the exposure assessment of population studies and to capture small spatiotemporal variations in levels of pollutant such as O_3_ (Briggs 2005; Jerrett et al. 2005; Zou et al. 2009). For instance, land-use regression (LUR) models are used to predict pollutant concentrations at unmonitored sites based on regression models of georeferenced covariates that predict observed (i.e., measured) data from monitored sites (Beelen et al. 2009; Jerrett et al. 2005). Kriging and the Bayesian maximum entropy (BME) framework are interpolation methods that assign a series of weights to observed monitoring station data to compute interpolated values of pollutants at unmonitored sites (Bell 2006; Bogaert et al. 2009; Christakos and Vyas 1998; de Nazelle et al. 2010).

The main objective of this work was to compare the accuracy of three spatiotemporal models to predict ground-level O_3_ in Quebec (Canada). We used a land-use, mixed-effects regression model developed with readily available data (air quality and meteorological monitoring data, road networks information, latitude) and two spatiotemporal interpolation models: *a*) a combined land-use BME model incorporating both O_3_ monitoring station data and the land-use mixed model outputs (BME-LUR), and *b*) a kriging method based only on available data from O_3_ monitoring stations (BME kriging).

## Methods

**Figure 1:**
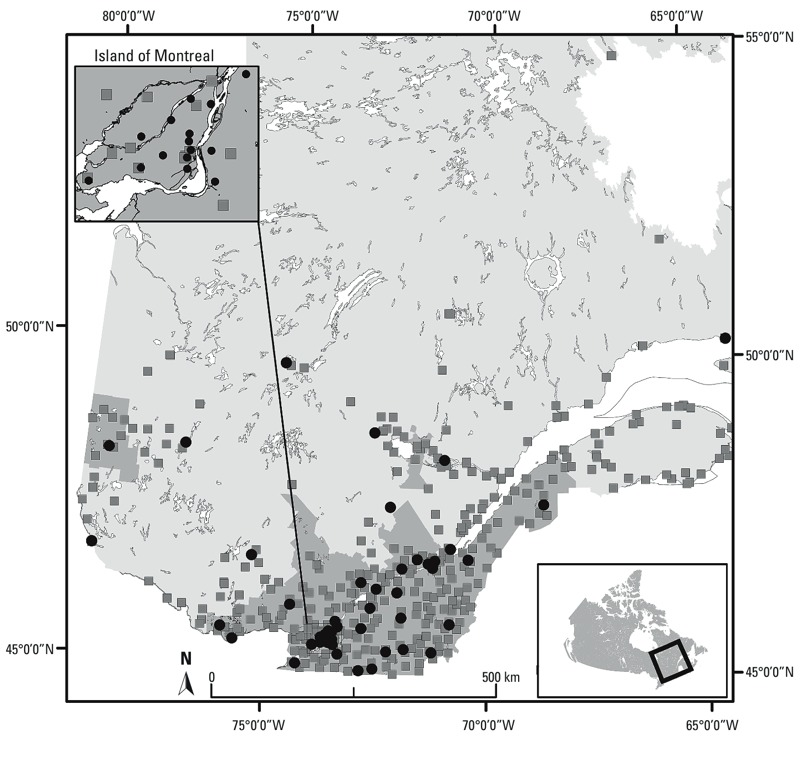
Geographical location of O_3_ monitoring (black circles) and meteorological stations (gray squares) in the study region (dark gray). Locations are for monitors used at any time during the study period.

*O_3_ monitoring data*. We retrieved hourly ground-level O_3_ observations for 1990 through 2009 from the National Air Pollutant Surveillance (NAPS) program (Environment Canada 2012) (Figure 1). We calculated only 8-hr midday (0900–1700 hours) O_3_ concentrations during the summer months (May through September) because O_3_ concentrations during the winter and at night are almost null in Quebec, and we included data for all available days with < 25% missing data (i.e., days with hourly data for at least 6 of the 8 hr).

In Quebec, the number of O_3_ monitoring stations increased from 2 stations in 1990 to a total of 50 stations available at the end of 2009. Up to 51 stations were available at some point in time during the period, resulting in 156,060 total observations (station-days). All stations had a limit of detection (LOD) of 1 ppb by 1995, and most stations had an LOD of 10 ppb before 1995. Measured 8-hr daily O_3_ levels were recorded as 0 ppb for 373 observations (station-days) during the study period, of which 355 observations were recorded before 1995, when less sensitive instruments were in use. However, these data were retained in our analyses because they represented only 0.02% of the observations used to develop the three models.

Road density data. We extracted road density data from the Digital Mapping Technology Inc. (DMTI) 2010 Road Layer Dataset (DMTI Canmap Streetfile version 2010.3; DMTI Spatial Inc., Markham, Ontario, Canada) and retained major roads, primary and secondary highways, and freeways from all road layers. We measured the total kilometers of such roads within a 1-km buffer around the O_3_ stations and the road density was expressed in km/πkm^2^ (i.e., kilometers of road within a circular area with a 1-km radius).

Meteorological data. We obtained meteorological data from the National Climatic Data and Information Archive of Environment Canada for May through September of 1990–2009 (Environment Canada 2011). We extracted mean 8-hr temperature (from 0900 to 1700 hours for days with ≥ 75% of the data available) and daily precipitation records for all weather stations in Quebec. Figure 1 shows the locations of all available meteorological stations.

*Development of models*. LUR mixed-effects model. We developed a linear LUR mixed-effects model to predict O_3_ concentrations measured at monitoring sites using R software version 3.0.1 (R Project for Statistical Computing; http://www.r-project.org/). The variables used in the model were temperature, precipitation, day of year, year, road density in a 1-km buffer area, and latitude. Temperature and precipitation data were from the weather station closest to each O_3_-monitoring site. We shifted and rescaled these variables to produce coefficients of a similar range and to render the intercept interpretable. Specifically, we subtracted 121 from the numeric day of the year to shift its range from 121–274 to 0–153, subtracted 1990 from the year to convert its range from 1990–2009 to 0–19, and subtracted 4,995.9 (the minimum value) from the latitude variable to standardize its range to 0–583.3 km (such that a latitude of 0 represents that latitude of the most southerly O_3_ monitoring station.)

We used linear splines to model temperature (one knot at 18°C), road density (one knot at 15 km/πkm^2^), and latitude (one knot at 50 km) because their relationships with O_3_ were not linear. We determined the number and location of the knots by visual inspection and selected linear splines over cubic splines to increase simplicity because the results were nearly as good (i.e., the root mean square difference between the prediction of the two models was < 0.81 ppb). Therefore, we represented associations with O_3_ by two model coefficients (one for each linear segment) for each of these variables.

We nested values within stations, which were treated as a random intercept. Thus, we estimated average 8-hr daily O_3_ concentrations for each observed station-day as follows:

O_3_ = β_0_ + β_1_*X*_low_temperature_ + β_2_*X*_high_temperature_ + β_3_*X*_precipitation_ + β_4_*X*_dayofyear_ + β_5_*X*_year_ + β_6_*X*_low_road_ + β_7_*X*_high_road_ + β_8_*X*_low_latitude_ + β_9_*X*_high_latitude_ + *u*_station_ + ε, [1]

where *X* is the value of the variable for that station day, β is the coefficient for that variable, *u* is the random effect associated to that station, and ε is the remaining error of the station day.

BME-LUR and BME kriging analysis. We developed both BME kriging and BME-LUR models for a territory involving census districts of population density > 5 people/km^2^ in 2006 (Statistics Canada 2007). This was to ensure that a large proportion of the Quebec population would be covered by the study area, without including areas with very low populations. We created a 50-km buffer around our present study area to avoid any edge effects caused by a lack of data just outside a census district. Therefore, the selected study region was situated between approximately 42°–50°N latitude and 65°–80°E longitude, encompassing a total area of 103,110 km^2^ (Figure 1).

The “hard” data we used to develop the BME kriging and BME-LUR models were the measured O_3_ concentration data provided by the O_3_ monitoring stations for all eligible station-days during 1990 to 2009. “Soft” data refers to information that can be used to improve estimates by compensating for the limited amount of measured data. Usually, soft information is based on some *a priori* knowledge of the physical processes that affect the spatiotemporal distribution of the pollutant. For our analysis, the soft data we used were O_3_ levels (and their respective normal errors) estimated from the land-use mixed-effects regression model for 1 km × 1 km grid cells within the study area for May–September 2005, the year used as the reference year for cross-validation.

Soft data from the LUR model was composed of an O_3_ estimate for each location and an associated error estimate. The error estimated for each modeled point (each center of the 1 km × 1 km grid cell) was the sum of the squares of the standard errors from the fixed effects and the square of the standard deviation of the soft random intercept. For the O_3_ estimate itself (soft data), only the fixed portion of the LUR model was used to create a value because the mean random effect was 0. There were a total of 278,633 possible grid points per day (approximately 42 million spatiotemporal points were possible overall), with the O_3_ levels estimated using data from the closest meteorological station. Soft data were estimated only when all predictors were available. It was impossible for a large portion (around 99%) of points to be estimated because of missing precipitation or temperature data at the closest monitor (mainly in the inhabited northern regions of our present study area). However, this did not influence the cross-validation analysis because that analysis was limited to the location of the O_3_ monitors that had sufficient soft data.

We treated kriging as a special case of the BME in which we used only hard data (i.e., station-days with O_3_ monitoring station data) without including soft data estimates from the LUR model, and thus we refer to this model as “BME kriging.” Because of the spatiotemporal nature of the model used, kriging in this instance refers to a spatiotemporal interpolation of O_3_, and not merely a spatial estimate. We implemented the BME-LUR and BME kriging analysis to estimate daily 8-hr average O_3_ levels at a 1-km^2^ grid using Matlab 2007 (MathWorks, Natick, MA, USA) and the SEKS-GUI program, version 0.69.5 (Yu et al. 2007).

To account for short-term and small-scale patterns in the O_3_ data and to remove any spatiotemporal autocorrelative patterns, we used a Gaussian detrending model (Yu et al. 2007) at a distance of 25 km and a temporal trend of 2 days. This detrending is used to facilitate the interpolation of the remaining stochastic structure of the data. Such detrending algorithms are common in spatial estimation techniques such as kriging. Although several detrending methods exist, the SEKS-GUI program provides the Gaussian detrending algorithm as its only detrending option. From visual inspection of time series of O_3_ levels at monitoring stations, and of spatial distributions of daily O_3_ levels across all stations, Gaussian detrending appeared to be a sufficient function to remove spatiotemporal trends. The detrended data was then used as our stochastic spatiotemporal data set for BME kriging and BME-LUR modeling.

Ozone soft and hard data was not normally distributed. We therefore corrected soft and hard data using *n*-scores normalization before analysis because a normal distribution is a necessary condition for accurate estimation by the BME (Yu et al. 2007). We constructed a spatiotemporal covariance model to describe the stochastic processes affecting O_3_ levels after localized detrending. We used the resulting model for estimating the O_3_ values, followed by denormalization and retrending of the estimated value.

*Cross-validation*. We performed cross-validation to test the predictive ability of the different models and to find the best predictive model. Cross-validation was performed using data from 2005 as a sample year. We did cross-validation for summer days at each monitoring station for which a LUR model estimate could be created (*n* = 3,986 station-days points among 30 stations). In BME-kriging and BME-LUR, we removed all hard data for up to 1 year before each cross-validation date at each monitoring station, for the cross-validation at that station. This was done to eliminate the effects of temporally near data. This approach allows for the assessment of the estimation accuracy in different space–time domains while avoiding the potentially biased interpretation of the estimation results induced by purely temporal autocorrelation (Yu et al. 2009). To perform our cross-validation, we removed a given station-day’s hard data and estimated it using the remainder of the data (i.e., leave-one-out validation). The soft data used for the cross-validation did contain the information from all stations (i.e., the station was not removed during the construction of the LUR) because removing individual stations from the leave-one-out analysis would have had a marginal effect on the construction of the LUR model and subsequent soft data [each station represents approximately 2% of the data (1/50 stations)].

We compared estimation errors (estimated values minus observations) across methods for each station-day versus the O_3_ values for that monitoring station at that time. We used root mean-square errors (RMSEs) to estimate the total magnitude of error. We also defined a percent change in mean square error (PCMSE) as used by de Nazelle et al. (2010), where the results correspond to the percent increased or decreased estimation accuracy of the O_3_ concentration prediction based on the LUR or BME kriging models compared with corresponding predictions based on the BME-LUR. We assessed visually for unusual spatial or temporal patterns in the distributions of the average estimated errors (estimated versus observed data).

Finally, we compared observed exceedances of the 8-hr Canadian Ambient Air Quality Standard (i.e., 65 ppb) identified using monitoring station data to exceedances identified using model estimates. To do so, we first transformed monitored and estimated O_3_ data variables into binary variables (0 = no exceedance, 1 = exceedance) and compared the estimated exceedances to the observed exceedances using Cohen’s kappa measure of agreement.

## Results

Table 1 presents the description of the data used for the development of the LUR model for the years 1990–2009. Predictors and O_3_ data were available at 39 O_3_ monitoring stations on 2,441 days. Because information was not available concurrently at all stations and all days, we used 29,685 spatiotemporal points (station-days) of 118,560 possibilities (152 days × 20 years × 39 stations) to develop the model. These 29,685 points were spatiotemporal moments for which we concurrently had information on O_3_ levels, temperature, and precipitation. Eight-hour O_3_ concentrations ranged from 0 to 104 ppb, 8-hr temperatures ranged from –3.5 to 33.9°C, daily precipitation ranged from 0 to 123.8 mm/day, and road density ranged from 0 to 25.4 km/πkm^2^. The range of latitude values was between 0 and 583.3 km.

**Table 1:** Descriptive statistics of variables used in developing the LUR model for 1990–2009.

Variable	No. of spatiotemporal points^*a*^	Mean ± SD	Minimum	Maximum
8-hr O_3_ concentration (ppb)	29,685	31.2 ± 13.1	0.0	104.0
8-hr temperature (°C)	29,685	19.1 ± 5.3	–3.5	33.9
Precipitation (mm/day)	29,685	3.0 ± 7.1	0.0	123.8
Road density (km/πkm^*2*^)	39	6.4 ± 7.9	0.0	25.4
Rescaled latitude (km)	39	114.6 ± 134.6	0	583.3
^***a***^We used 29,685 of 118,560 possible station-days (limited by temperature and precipitation variables).				

The LUR model is summarized in Table 2. Considering the estimated effect size (see footnote of Table 2 for clarification on the calculation) of each variable, temperature, day of the year, and road density were the main predictors. In this model, coefficients for linear spline functions of temperature (≤ 18°C and > 18°C) were positively associated with O_3_ concentrations, whereas precipitation, day of the year, year, and coefficients for linear spline functions of low and high road density and of low latitude (< 50 km) were negatively associated with O_3_ levels. Overall, all predictors had a significant association, except the coefficient of the linear spline function for high latitude. To better visualize the fixed effects, see the LOESS plots of bivariate relationships of these predictor variables in Supplemental Material, Figure S1. Every coefficient of the LUR model was in agreement with the LOESS plots, and with known processes of the formation and the destruction of O_3_, except for cold temperature. Based on the LOESS plot, we expected temperatures between –3.5°C and 18°C to have no relation with O_3_, or the relation to be slightly negative, whereas in the LUR model, after controlling for latitude, year and day of the year, the relation between O_3_ and the lowest temperatures was slightly positive.

**Table 2:** Summary of the LUR model for O_3_ concentrations in the region of study (1990–2009).*^a^*

Fixed effect	Coefficient	SE	Effect size^*c*^
Constant	39.530	1.577	—
Temperature ≤ 18ºC^*b*^	0.218	0.021	39.461
Temperature > 18ºC^*b*^	2.139	0.019	—
Precipitation	–0.010	0.001	–1.238
Day of the year	–0.107	0.001	16.371
Year	–0.165	0.018	3.315
Road density ≤ 15 km/πkm^2^^*b*^	–0.255	0.098	–14.995
Road density > 15 km/πkm^2^^*b*^	–1.074	0.219	—
Latitude ≤ 50 km^*b*^	–0.123	0.038	1.687
Latitude > 50 km^*b*^	0.003	0.003	—
^***a***^For the random effect, the SD of intercept is 2.464 (95% CI: 1.915, 3.170); the SD of residuals of mixed model is 8.904. ^***b***^Variables modeled as linear spline functions to account for nonlinear relations with O_3_. ^***c***^The effect size was calculated by β_*i*_*V*_*iMax*_ – β_*i*_*V*_*iMin*_ for non-splined variables, and by β_*iLower*_*V*_*iSpline*_ – β_*iLower*_*V*_*iMin*_ + β_*iUpper*_(*V*_*iMax*_ – *V*_*iSpline*_), where *V*_*iSpline*_ is the value of the knot of the variable of interest, β_*iLower*_ the coefficient for values lower than the knot value, and β_*iUpper*_ the coefficient for values greater than the knot value.			

Table 3 describes the hard and soft data used to build the BME-LUR and BME kriging models. Hard data were observations at monitoring sites for 1990–2009 (*n* = 103,669 of 156,060 station-days with O_3_ data), and predicted soft data estimates were derived from the fixed effect portion of the LUR model and errors estimated from the fixed and random effects of the same model for the year 2005 only (152 days). Therefore, we could estimate 90,847 spatiotemporal points from the LUR model, considering the availability of temperature and precipitation information concurrently, of around 42 million maximum possible spatiotemporal points (152 days × 278,633 possible grids points per day in the present study area). For BME kriging and BME-LUR, we used the same detrending and covariance structures to describe the spatiotemporal covariance pattern in the data. The covariance model used to fit the measured spatiotemporal covariance of the data consisted of two components: a short-term (2-day exponential) long-distance (100-km exponential) trend that described the majority of the variability (covariance = 0.9), and a second component (covariance = 0.1) describing the weekly (3-day cosinusoidal) trend in covariance in time with a small spatial (i.e., local 12.5-km exponential) scale because of the cyclic nature of O_3_ in urban stations in Quebec, where O_3_ tends to be lower on the weekends and to rise during weekdays. Modeled covariances as derived from the information above are presented in Supplemental Material, Figure S2.

**Table 3:** Statistics for measured (hard) O_3_ data (1990–2009) and predicted and error “soft” data from the LUR (year 2005) used for BME-LUR and BME kriging models.

Variables	No. of spatiotemporal points	Mean ± SD (ppb)	Minimum (ppb)	Maximum (ppb)
Hard data (*n* = 51)	103,669^*a*^	30.6 ± 12.5	0.0	110
Soft data at a 1-km grid (predicted)	90,847^*b*^	46.3 ± 9.3	12.1	76.4
Soft data (error)	90,847^*b*^	6.9 ± 1.8	5.5	63.9
^***a***^103,669 of 156,060 station-days with O_3_ data (limited by O_3_ data availability only) as hard data for BME-LUR and BME kriging models. ^***b***^We estimated 90,847 spatiotemporal points with data for temperature and precipitation as soft data for 2005 of approximately 42 million maximum possible spatiotemporal points (152 days × 278,633 possible grid points per day in the area of the present study).				

Table 4 describes the cross-validation results for the three models for the year 2005 at the 30 stations available to produce the soft data with all mixed model predictors (*n* = 3,980). For the BME-LUR, on 25 June, estimates at six stations, all located in the southeastern portion of the study area, could not be estimated with the BME-LUR. On that day, all measurements at these stations were high (hard data) (75–78 ppb) when compared with the range of values of the calculated soft data (28–48 ± 6.6 ppb) for that day. Overall, the BME-LUR was the most predictive model (*R*^2^ = 0.653), and had the lowest RMSE (7.06 ppb). The LUR model performed better and with greater precision (*R*^2^ = 0.466, RMSE = 8.747) than the BME kriging model (*R*^2^ = 0.414, RMSE = 9.164). The BME-LUR outperformed the LUR model and BME kriging by 19.9% and 23.0% using PCMSE, respectively. Finally, the Cohen’s kappa of the BME-LUR (*n* = 18 predicted exceedances; kappa = 0.525, 95% CI: 0.495, 0.555) obtained from the comparison of 8-hr Canadian Ambient Air Quality Standard (65 ppb) monitored (*n* = 34 observed exceedances) and estimated concentrations suggests moderately good agreement between the model and the measurements. The BME-LUR outperformed both BME-kriging (*n* = 39 predicted exceedances; kappa = 0.169, 95% CI: 0.138, 0.200) and the LUR model (kappa = 0 because no predicted value was > 65 ppb).

**Table 4:** LUR, BME kriging, and BME-LUR models leave-one-station-out cross-validation results for year 2005 [*n* = 30 O_3_ monitoring stations (3,980 estimated points)].

Method	*R*^2^	RMSE (ppb)	PCMSE
LUR	0.466	8.747	–19.9%
BME kriging	0.414	9.164	–23.0%
BME-LUR	0.653	7.057	—

**Figure 2:**
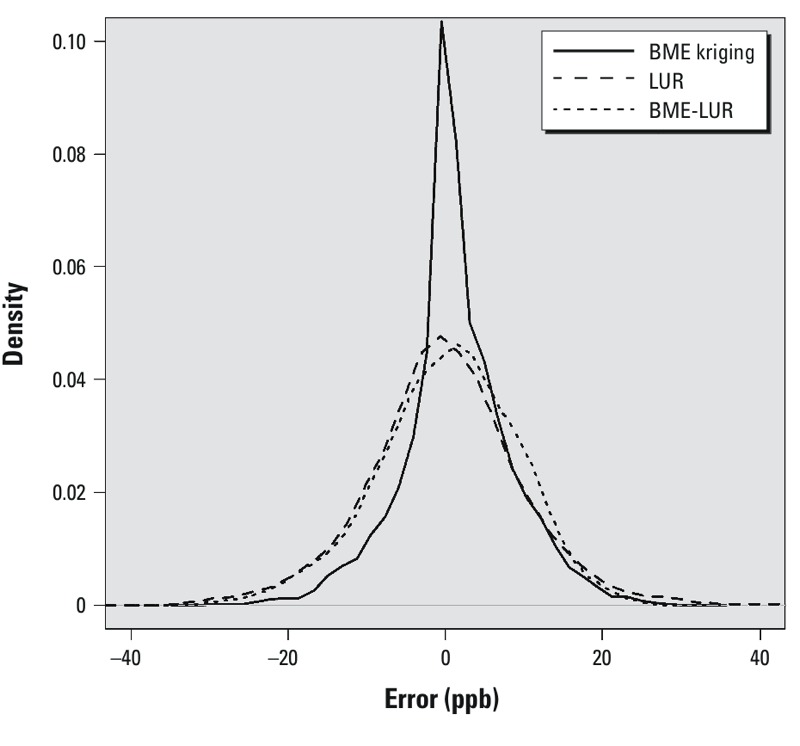
O_3_ mapping error estimates (RMSEs) from the leave-one-station-out cross-validation [where error = estimated – measured (observed) O_3_ concentration (in ppb) at each monitoring station] based on the LUR (mean ± SD; 0.282 ± 8.93 ppb), BME kriging (0.130 ± 9.804 ppb), and BME-LUR (1.339 ± 7.086 ppb) models for the year 2005.

**Figure 3:**
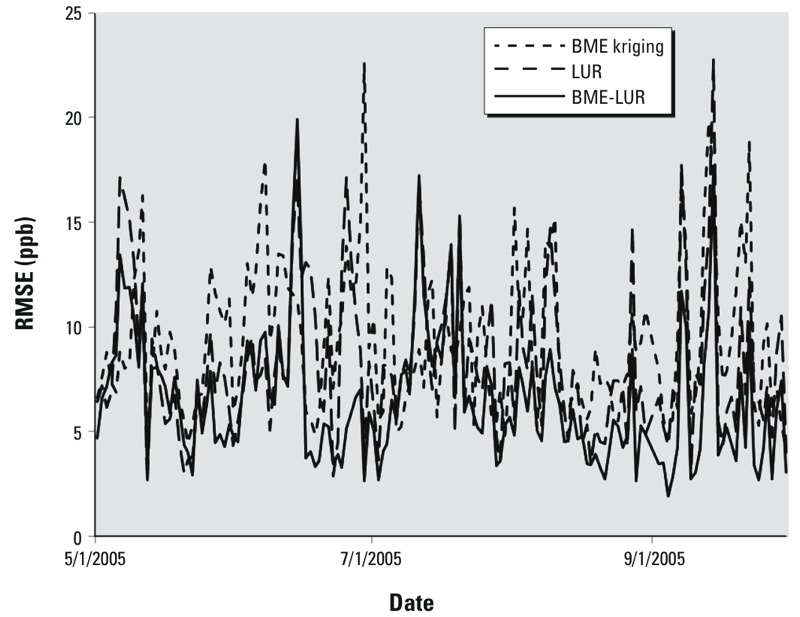
Mean temporal O_3_ error estimates (RMSEs) based on the leave-one-station-out cross-validation for LUR, BME kriging, and BME-LUR models for the year 2005.

**Figure 4:**
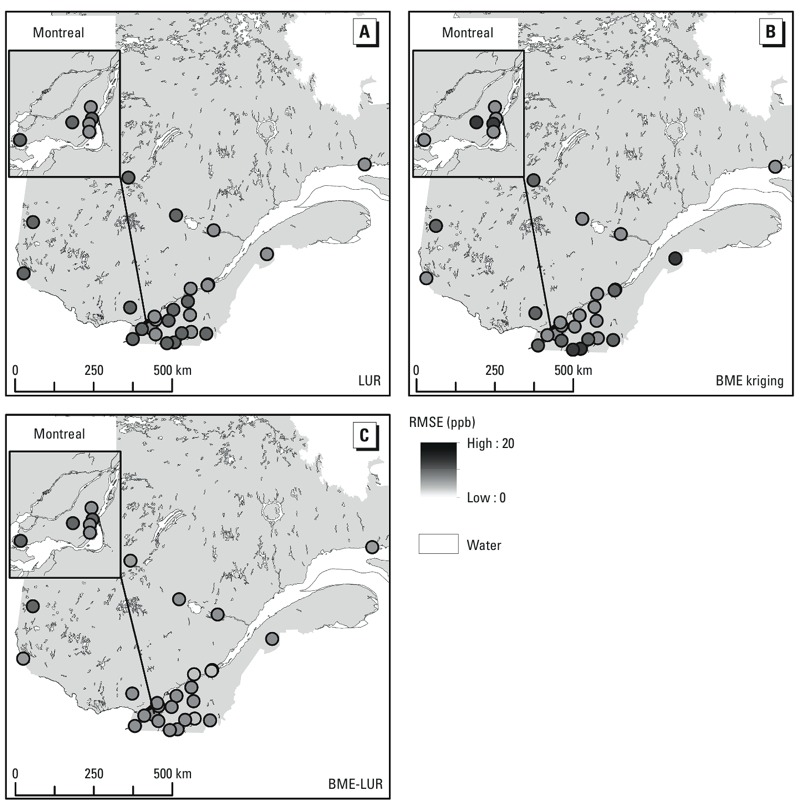
Spatial distribution of mean O_3_ error estimates (RMSEs) in the study area (year 2005) based on the leave-one-station-out cross-validation for LUR (*A*), BME kriging (*B*), and BME-LUR (*C*) models.

**Figure 5:**
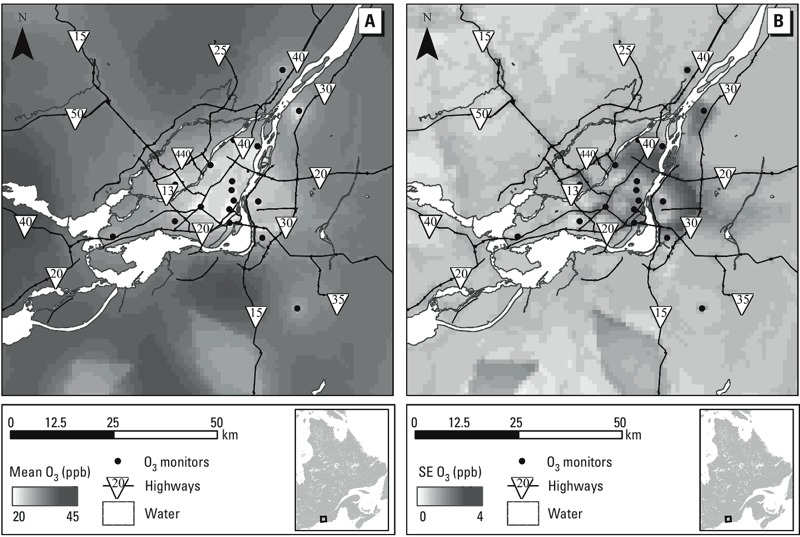
Mean O_3_ levels (0900–1700 hours) (*A*) and SEs (*B*) predicted from the BME-LUR at a 1-km grid across the greater Montreal region in Quebec (Canada) for the summers of 2006–2009.

A graph of the distribution of errors in the O_3_ concentration estimates generated by each model (i.e., the difference between estimated and observed values) based on the leave-one-out analysis also demonstrated that the BME-LUR was the more accurate model (Figure 2). As shown in Figure 3, the RMSE of the three models appears to be stochastic in time. Figure 4 shows that the RMSE of the BME-LUR in space (at all stations) was closest to zero in comparison with BME-kriging and the LUR. Figure 5 represents a map of predicted mean daily O_3_ levels (0900–1700 hours) and SEs at a 1-km grid across the greater Montreal region for the summers of 2006–2009. Levels of O_3_ are higher around the suburbs of Montreal compared with downtown metropolitan areas and concentrations are also greater in places far from highways (Figure 5A). Moreover, a greater difference between observed and estimated O_3_ concentrations may be found in the northeast of the greater Montreal region (Figure 5B).

## Discussion

Overall, our findings suggest that error of estimation in the interpolation of O_3_ concentrations using the BME method may be improved with the inclusion of a LUR model developed with a readily available database.

We found that the estimation of O_3_ across monitoring sites was more accurate with the BME-LUR model compared with other models; this difference was close to 20% in *R*^2^ and around 2 ppb in RMSE. These results are consistent with previous work. For instance, Yu et al. (2009) modeled air pollutant concentrations in North and South Carolina (USA) and found that the integration of soft information by the BME method effectively increased the estimation accuracy for O_3_ predictions compared with estimates derived using BME kriging. Yu et al. (2009) used measurements from monitoring stations as soft data, whereas we created soft data from outputs of a LUR model. Yu et al. (2009) did not report the *R*^2^ and RMSE values, but the mean and SD of their estimation errors for daily estimates were similar to ours in the present study (Yu et al.: kriging = 0.483 ± 7.035 and BME = 0.177 ± 6.845 ppm; present study: kriging = 0.414 ± 9.164 and BME-LUR = 0.653 ± 7.057). de Nazelle et al. (2010) also found better predictive accuracy for the representation of space-time O_3_ distribution in North Carolina with a BME model based on observed (hard) and modeled (soft) data from a stochastic analysis of an urban-intercontinental-scale atmospheric chemistry transport model, compared with kriging method estimates based on hard data only. We found that, similar to de Nazelle et al. (2010), O_3_ estimates for areas farther away from monitoring stations were more accurate when soft data was used in the BME versus kriging alone. As in our work, their PCMSE values were always negative (between –1.486 and –27.699, depending on the cross-validation radii of exclusion points), indicating that the integration of observed and modeled prediction was consistently more accurate than relying solely on observations. Furthermore, agreement between modeled and observed Canadian Ambient Air Quality Standard exceedances was highest for estimates based on the BME-LUR.

We found that error estimates from the BME-LUR model were more accurate when monitoring stations were clustered in the region of the study, such as in the southern (i.e., more urban) part of Quebec (Figure 4). This result is consistent with results of Yu et al. (2009), which indicated that the locations where the estimates exhibit higher discrepancies from the data values were mostly close to regions of data scarcity.

The LUR model was slightly more accurate (lower RMSE) than the BME kriging model (Table 4). Coefficients of the LUR model indicated that linear spline functions of temperature were positively associated with O_3_ concentrations, whereas precipitation, day of the year, year, and coefficients for linear spline functions of low and high road density and of low latitude were negatively associated with O_3_ levels (Table 2). The LUR model coefficients for the spline temperature variable are in line with the expected trend (U.S. EPA 2006) and suggest an increase of O_3_ with temperature, which is more pronounced at higher temperatures. With regard to road density, both coefficients for linear spline functions of low and high density were negative, and this may be explained by the fact that at the regional scale, low traffic represents lower concentrations of O_3_ precursors [traffic-related pollutants such as nitrogen oxides (NO_x_)], whereas at the local scale, low traffic represents lower destruction of O_3_. The other fixed effects of the LUR model are also in agreement with the known atmospheric processes of O_3_ and highlight that its formation rely on various factors such as sunlight. Ozone concentrations are also greater with altitude and show diurnal and weekly variations with higher levels during weekdays (Finlayson-Pitts and Pitts 1997; U.S. EPA 2006). Finally, the negative coefficient found for day of the year variable highlight the small intra-annual decrease in O_3_ levels from May to September in Quebec.

Nevertheless, the fact that the LUR model was slightly more accurate than BME kriging is inconsistent with what was found by Beelen et al. (2009), who developed maps of O_3_ levels across the European Union using a regression model with altitude, distance to sea, major roads, high-density residential areas, and a combination of meteorological data as predictors. They obtained values of *R*^2^ = 0.54/0.38 and RMSE = 8.63/8.74 ppb respectively for the regression and kriging models at rural scale. At urban locations, kriging was more accurate than the regression model with only the high-density residential predictor (regression/kriging: *R*^2^ = 0.38/0.61 and RMSE = 7.32/5.84). Kriging methods predict well when a dense and representative monitoring network is available (Briggs 2005; Jerrett et al. 2005; Laslett 1994). In the present study, BME-LUR was more accurate in estimating O_3_ levels than LUR and BME kriging at urban and suburban scales (i.e., island of Montreal and its surrounding area), and LUR was more accurate than BME kriging in urban areas only (Figure 4). In Quebec, the monitoring station network is relatively sparse and the good correlations between the predictors used in the LUR model and the measured O_3_ concentrations at monitoring stations may at least partially explain the relatively weak performance of BME kriging.

We created maps representing mean O_3_ levels (0900–1700 hours) and SE predictions from the BME-LUR at a 1-km grid for the summers of 2006–2009 to visualize how the model would estimate O_3_ in urban and suburban areas of the greater Montreal region. As observed in Figure 5, levels of O_3_ are higher around Montreal Island (suburban areas) compared with downtown metropolitan (central Montreal Island) areas, and concentrations are also greater in areas far from highways. This may be explained by the fact that the efficiency of O_3_ production depends on NO_x_ concentrations. In areas with low NO_x_ concentrations (e.g., in rural areas), O_3_ production increases with higher levels of NO_x_. In downtown metropolitan areas where the highest NO_x_ concentrations may be found, a net destruction of O_3_ by reaction with NO (nitrogen monoxide) has been reported (U.S. EPA 2006). Also, we found a greater difference between observed and estimated O_3_ concentrations in the northeast of the greater Montreal as indicated by Figure 5B, and this may be explained by the possible incongruity between soft and hard data points, hard data points themselves, or by a possible lack of O_3_ stations outside the Montreal area.

As mentioned previously, 6 stations could not be computed with the BME-LUR on June 25th. In-depth analysis reveals that all these stations had high monitored values (hard data) when compared with the range of values of the calculated soft data for that day. To our knowledge, this issue has not been reported elsewhere in the literature and investigations of BME estimation failure should be realized in future studies.

The developed BME-LUR model presents other limitations. For instance, the meteorological variables, temperature and precipitation, used to estimate soft data do not represent the complete atmospheric processes of O_3_. This would have been more correctly assessed with the use of some integrated meteorology models such as the CMAQ (Community Multiscale Air Quality) modeling system. However, such models do not capture small area estimations such as our LUR model predictions (U.S. EPA 2012). Another limitation is that the LUR model predictions were only estimated for each 1-km grid of the territory because of computational constraints, as adding soft data at 100-m resolution would have dramatically increased the amount of time needed to run the BME-LUR. Computational time required to create maps is another limitation. In the present study, 90 days were needed to create maps of O_3_ levels for an area of 103,110 km^2^ at a resolution of 1 km while running multiple processors on a high-powered computer (2.93 GHz 4-core processor and eight concurrent threads with 6 GB RAM). This computational time can be improved by reducing the resolution of the study area or the number of soft data points, as well as by estimating only points of interest (e.g., residential addresses of interest vs. a 1-km grid).

Despite the computational demands, the BME-LUR adds value to the O_3_ exposure estimation because it generates the complete probability distribution of exposure at each point in space and time (Yu et al. 2007) and it reduces the estimation errors. This may lead to less biased effect measures and greater statistical power in health studies (Baker and Nieuwenhuijsen 2008; Briggs 2005; Goldman et al. 2012).

For implementation in future health studies, the BME-LUR might be improved by including additional predictors in the LUR model, such as population density, land use, topography, and industrial sources of precursors (Hoek et al. 2008). As noted by Beelen et al. (2009), stratification of the study area (e.g., separating urban and rural areas) could also improve model predictions.

## Conclusions

We aimed at comparing the ability of three spatiotemporal models to predict ground-level O_3_ in Quebec (Canada) to improve O_3_ health risks assessment. The BME-LUR model appeared to be the best model for exposure prediction. This work illustrated the accuracy of the BME-LUR models to predict air pollutants such as O_3_ across space and time over LUR and BME kriging methods and that error of estimation in the interpolation of O_3_ concentrations can be greatly reduced using outputs from a LUR model that can be developed with readily available data.

## Supplemental Material

(264 KB) PDF
