# Supplementary material for: Spatiotemporal Modeling of Ozone Levels in Quebec (Canada): A Comparison of Kriging, Land-Use Regression (LUR), and Combined Bayesian Maximum Entropy–LUR Approaches
Source: Environ Health Perspect. 2014 May 30;122(9):970–6. doi: 10.1289/ehp.1306566 (PMC4153742; doi:10.1289/ehp.1306566)
Supplement: (264 KB) PDF [file ehp.1306566.s001.pdf]

Environmental Health Perspectives doi:10.1289/ehp.1306566

Supplemental Material

Spatiotemporal Modeling of Ozone Levels in Quebec (Canada): A Comparison of Kriging, Land-Use Regression (LUR), and Combined Bayesian Maximum Entropy–LUR Approaches

Ariane Adam-Poupart, Allan Brand, Michel Fournier, Michael Jerrett, and Audrey Smargiassi

Figure S1A: Temperature VS Ozone

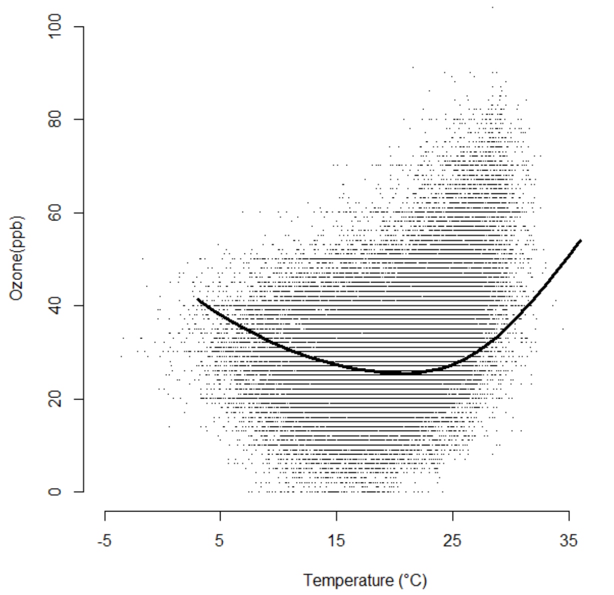

Figure S1B: Precipitation VS Ozone

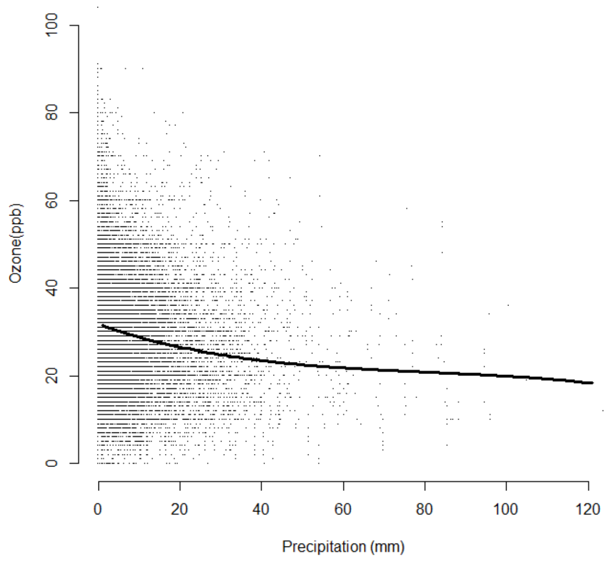

Figure S1C: Day of year VS Ozone

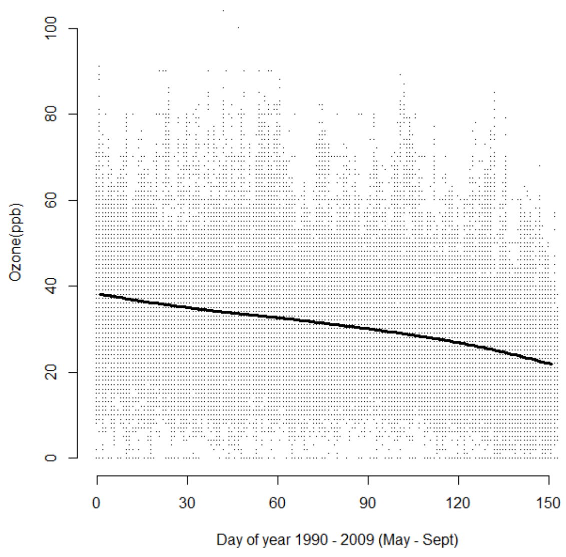

Figure S1D: Year VS Ozone

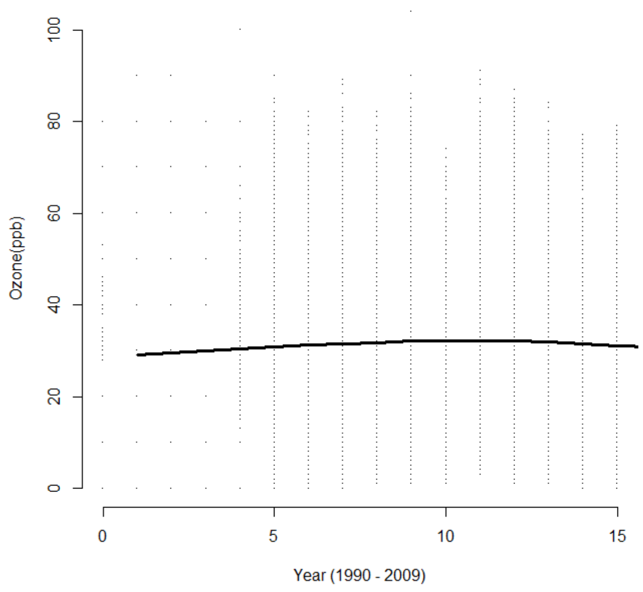

Figure S1E: Road density VS Ozone

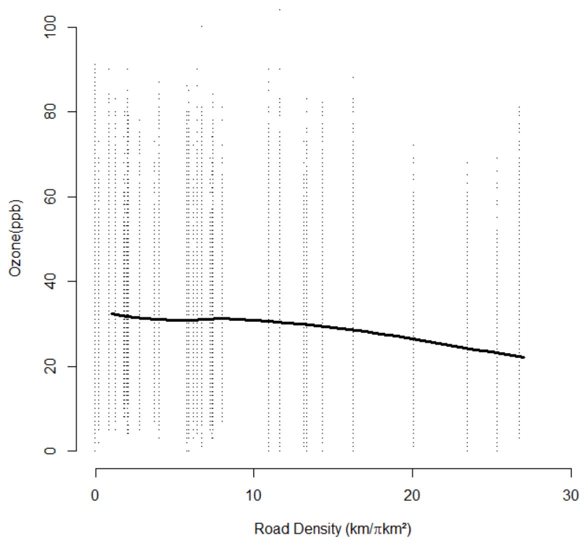

Figure S1F: Latitude VS Ozone

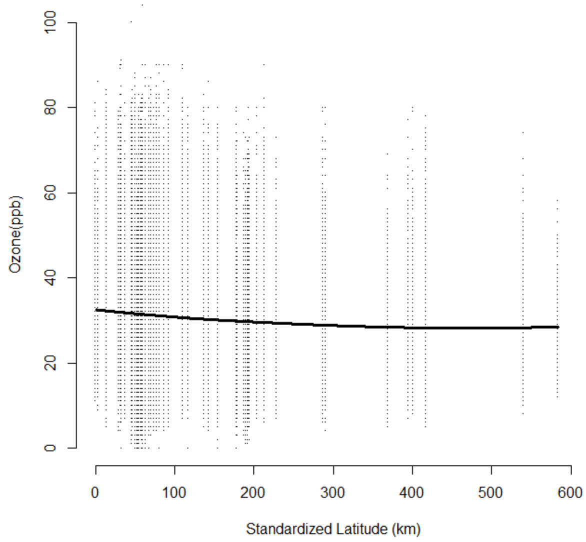

Figure S1. LOESS plots for each bivariate relation between predictor variables of the LUR model and ozone levels.

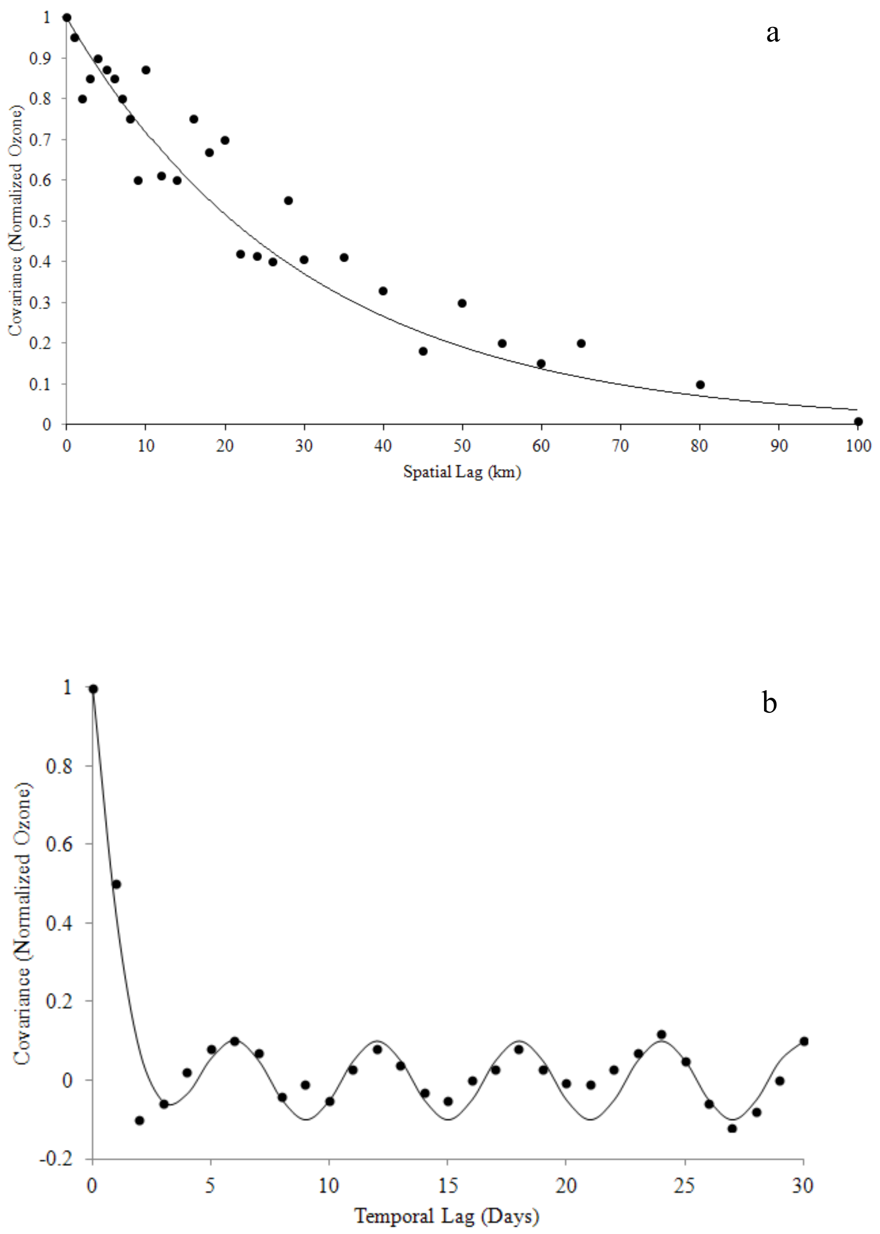

Figure S2. Spatial (a; at temporal lag = 0 days) and temporal (b; at spatial lag = 0 km) covariance plots of de-trended normalized ozone data from the BME kriging model used by the BME kriging and BME-LUR models.
